# Antimicrobial resistance profiles and genetic basis of resistance among non-fastidious Gram-negative bacteria recovered from ready-to-eat foods in Kibera informal housing in Nairobi, Kenya

**DOI:** 10.1099/acmi.0.000236

**Published:** 2021-06-11

**Authors:** John Maina, Perpetual Ndung’u, Anne Muigai, John Kiiru

**Affiliations:** ^1^​ Center for Microbiology Research, Kenya Medical Research Institute, Nairobi, Kenya; ^2^​ Jomo Kenyatta University of Agriculture and Technology, Kenya

**Keywords:** extended-spectrum β-lactamases (ESBLs), Kibera informal housing, multidrug-resistant strains (MDR), microbial food contamination, antimicrobial resistance genes (ARG), antimicrobial resistance (AMR)

## Abstract

**Objective:**

This cross-sectional study conducted in Kibera, Kenya, sought to gain insights on relative microbial contamination levels of popular unprocessed food types, determine antimicrobial resistance (AMR) burden and the carriage of integrons that are essential elements for spreading antimicrobial resistance genes (ARG). Foods analysed consisted of cooked vegetables (kale, cabbage, and nightshades), boiled cereal foods (beans, rice, and Githeri, which is a mixture of beans and maize), meat, Omena fish (fried silver cyprinids), and Ugali (a product of simmered maize flour in boiled water).

**Results:**

The analysis detected contamination levels exceeding 2×10^4^ c.f.u. ml^−1^ in 106 (38 %) of the 281 ready-to-eat foods analysed. The majority of food types had microbial contaminations of between 4.0×10^4^ and 2.3×10^6^ c.f.u. ml^−1^. Kale was the most contaminated with a mean of 2.3×10^6^ c.f.u. ml^−1^, while Omena was the least contaminated with 4.0×10^4^ c.f.u. ml^−1^. Foods sold close to open sewage and refuse sites were more contaminated than those sold in relatively ‘cleaner’ settings (*P* <0.0001, O.R 0.1162, C.I 0.1162–0.120). A total of 405 bacterial isolates were recovered and included; *Klebsiella* spp 116 (29 %), *Escherichia coli* 104 (26 %), *Enterobacter agglomerans* 88 (22 %), *Proteus mirabilis* 30 (7 %), *Salmonella* spp 28 (7 %), *Citrobacter freundii* 27 (7 %) and *Serratia marcescens* 12 (3 %). Imipenem (IPM, 100 %) was the most effective antimicrobial agent, followed by cefepime (98 %). Ampicillin (AMP, 33 %), trimethoprim (TMP, 27 %), and sulfamethoxazole (SMX, 23 %) on the other hand, were the least effective antimicrobials. The analysis also found ten isolates (2 %) that had co-resistance to third-generation cephalosporins, fluoroquinolone (CIP), quinolones (NAL) and aminoglycosides (GEN); hereby we refer to this phenotype as the βFQA. The prevalence of multidrug-resistant (MDR) strains was 23 % (93), while that of extended-spectrum β-lactamases (ESBL) producing strains was 4 % (17). The *bla*_TEM_ was the most prevalent (55 %) β-lactamase (*bla*) gene among the screened 93 MDR-strains. Carriage of class one integrons (*intI*1) was more common (23 %) than *intl*2 (3 %) among these MDR-strains. Bacterial diversity analysis using the GTG_5_-PCR found no significant clusters for analysed *E. coli* and *K. pneumoniae,* suggesting recovered isolates were genetically diverse and not due to non-clonal expansion. The findings of this study are an indication that contaminated foods can be a reservoir for enteric pathogens and a source of AMR strains.

## Background

The World Health Organization (WHO) estimates that more than 600 million persons worldwide suffer foodborne infections every year, and 420 000 die as a result. Human food may be contaminated at the farm, mainly when unsafe irrigation water is used, at various points in the supply chain, during processing, or due to unhygienic handling [1, 2]. Foodborne diseases cause massive economic losses. The World Bank estimates a total productivity loss of $95.2 billion and an additional $110 billion in treatment annually only in low and middle-income countries [3]. The African continent is most affected by these foodborne diseases partially due to poor urban sanitation. Informal housing characterized by poor planning and lack of proper sanitation infrastructures such as sewage drainage, inadequate clean water supply, poor hygiene, and environmental pollution are the most affected.

Although food contamination includes chemical substances, parasites, and viruses, bacterial contaminants and their toxins are responsible for most foodborne illnesses [4]. Enteric bacteria, in contrast to Gram-positive bacteria, have so far been the most prevalent causes of foodborne illnesses, perhaps due to their abundance in the environment and faecal contamination (WHO fact-sheet/food-safety, 2020). This microbial contamination is higher among ready-to-eat foods sold in many informal housing streets due to unhygienic preparing and serving environments. These street foods are particularly popular in many informal housing areas in Sub-Saharan Africa, partially due to their affordability and retail convenience [5]. However, many of these food-vending points have an open space layout and are often close to refuse sites and over-flowing sewage that further poses a risk of cross-contamination. Unsafe waste disposal, dust, prolonged storage of cooked food without proper refrigeration, and food exposure to vectors also act as possible contamination sources [6]. Therefore, there is a high possibility that foods consumed in such settings are contaminated with microbial agents that may emanate from the immediate surrounding environment. In resource-poor countries, simple fingerprinting methods such as the GTG_5_ PCR can be used to assess possible cross-contamination of ready-to-eat unprocessed foods with bacterial contaminates. Although this technique is not as sensitive as those based on whole-genome sequencing and analysis, it gives a preview of possible genetic diversity of bacterial population sets, predicting outbreaks and expansion of significant clones.

The gradual increase of foodborne infections has led to heavy antibiotic reliance across the globe, leading to antimicrobial resistance build-up in previously sensitive bacterial strains [7]. Foodborne pathogens such as *Salmonella* spp are increasingly becoming resistant to broad-spectrum antimicrobial agents such as cephalosporins (third- and fourth-generation), aminoglycosides (Gentamicin), and fluoroquinolone (Ciprofloxacin) [8]. These three classes of antimicrobial agents are widely used in Kenya to treat infections, especially in hospital settings. Therefore, co-resistance to β-lactams, aminoglycosides, and fluoroquinolone in bacterial strains (hereby referred to as the βFQA phenotype) reduces treatment options leading to poor treatment outcomes. The heavy usage of human medicine in animal feed production, veterinary, and agriculture have also been determined to be sources of emergence and spread of antimicrobial-resistant bacterial strains [9, 10]. Antimicrobial resistance, especially among Gram-negative bacteria, is also facilitated by exchanging resistance genes within and across species. This exchange is predominantly mediated by horizontal transfer of genes carried on integrons that may, in turn, be carried on conjugative plasmids. These integrons are gene capture systems in the form of resistance gene cassettes and can accumulate and disseminate antimicrobial resistance genes [11]. Although more than nine integron classes have been documented to date, classes one and two have widely been heavily implicated in antimicrobial resistance (AMR) [12, 13]. Integrons have been documented in clinical settings and animal isolates in Kenya, but little is known about carriage in foodborne isolates [14–16]. Furthermore, data on food contamination and the diversity of bacterial species recoverable from street foods remains scarce in Kenya. Surveillance of food contamination and bacterial strains' resistance profiles is essential in food safety and contamination prevention measures.

The current study sought to determine the diversity of non-fastidious Gram-negative bacteria, common etiological agents of foodborne infections, and establish their associated antimicrobial resistance patterns. The study also analysed the carriage of β-lactamases genes and possible clonal relatedness of isolates recovered from different ready-to-eat foods in a slum setting. Microbial contamination and diversity data in ready-to-eat street foods have the potential advantages of forming the basis for launching antimicrobial resistance surveillance systems. This could help in the early detection of foodborne outbreaks and identify possible hotspots where multidrug-resistant strains may arise and cause outbreaks that could be difficult to treat.

## Methodology

### Study site

This cross-sectional study was conducted in Kibera, informal housing located in the south west of Nairobi Central Business District. Kibera is the most extensive informal housing in Kenya, with a population of close to a million, with the vast majority living below the poverty line. These informal housings have poor sanitation infrastructure characterized by poor sewage drainage, uncollected refuse, and chronic water shortages. Most street foods sold in this slum are prepared and served close to open and burst sewers, potentially putting consumers at risk of foodborne infections.

### Study approval

Ethical clearance was obtained before the study commenced from the scientific ethical review unit of Kenya Medical Research Institute (KEMRI SERU), approval number KEMRI/SERU/CMR/POOO55/3514 [17].

### Sample collection

**Fig. 1.**
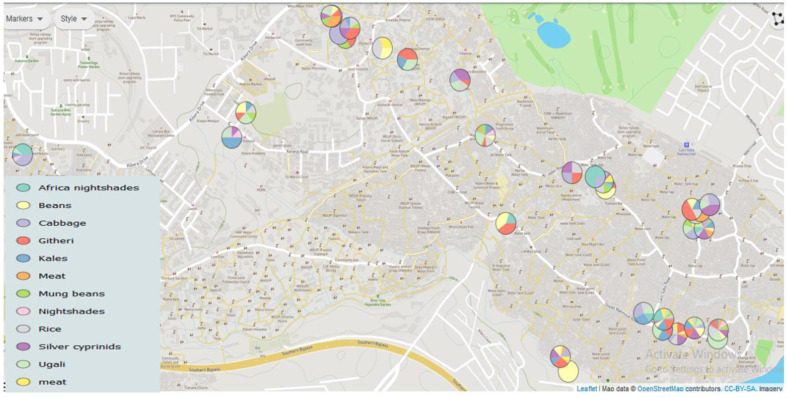
Distribution of ready-to-eat food sampling points in Kibera informal housings. This Kibera informal settlement map shows sampling points of ready-to-eat unprocessed foods. The sites were selected across various villages in Kibera slums based on location and hygiene characteristics. The map shows that most of these vending points are more concentrated at populated sections of the informal housings where consumer’s availability is high. This has constrained available resources leading to chronic water shortages and accumulation of refuse that is close to the vending points (within 10 metres radius). It was also noted that these vending points are also very close to open sewage.

This study obtained 281 ready-to-eat unprocessed food samples in Kibera informal housings between July to November 2017. A convenient sampling strategy was applied and involved randomly selecting vending points approximately 50 metres apart. Sample collection was done across all 13 villages that make up Kibera's informal settlements to ensure proper representation. Foods collected in this study were cooked vegetables (kales, cabbage, and nightshades) and boiled cereal foods (beans, rice, and Githeri, which is a mixture of beans and maize). Other food samples collected include Omena fish (fried silver cyprinids, meat and Ugali (a product of simmered maize flour in boiled water). About 25 g ml^−1^ of a food sample was collected using a sterile spoon, put in a sterile container, and transported in a cool box to the laboratory within 3 h. The global positioning system (GPS) coordinates of each sampled site were collected, and MDR hotspots mapping was done using the Micro-react online tool, Fig. 1.

### Sample processing

Microbial food contamination levels were assessed using the Enterobacteriaceae-specific 3M Petri film plates (Petrifilm Agua, USA). A 10 g ml^−1^ of food specimen was added into a stomacher bag containing 90 ml of sterile normal saline (0.85 % sodium hydroxide from Sigma-Aldrich), then homogenized for 1 min using a stomacher machine (Seward). A 1 ml of the homogenate was then serial diluted six-fold. An aliquot of 0.1 ml from the sixth dilution was spread on a 3M Petri-film plate and incubation done aerobically for 24–48 h at 37 °C.

For bacterial isolation, 0.1 µl of the homogenized sample was plated directly onto MacConkey (Oxoid, UK) and Eosin Methylene Blue Agar (Oxoid, UK), and incubation was done at 37 °C for 24 h. Non-duplicate isolates were then subjected to Gram-staining and biochemical test panels that included triple sugar agar iron, citrate, urea, and sulphur indole motility tests for bacterial species identification [18].

### Microbial food enumeration analysis

After 24–48 h of incubation, Enterobacteriaceae colonies on 3M Petri film (Petrifilm Agua, USA) were counted and microbial contamination levels expressed in colony-forming units per millilitre (c.f.u. ml^−1^):

c.f.u. ml^−1^=colonies counted × 0.1 ml of 10^−6^ dilution in 1 ml of the original sample.

Microbial contamination levels were classified according to guidelines stated by the hazard analysis and critical control point system (HACCP) by the Food and Agriculture Organization (FAO). A microbial contamination level of ≤10^2^ c.f.u. ml^−1^ was considered acceptable for human consumption. In contrast, foods with a 10^3^ c.f.u. ml^−1^ value and beyond were deemed to be unacceptable for human consumption [19].

### Antimicrobial susceptibility testing

**Fig. 2.**
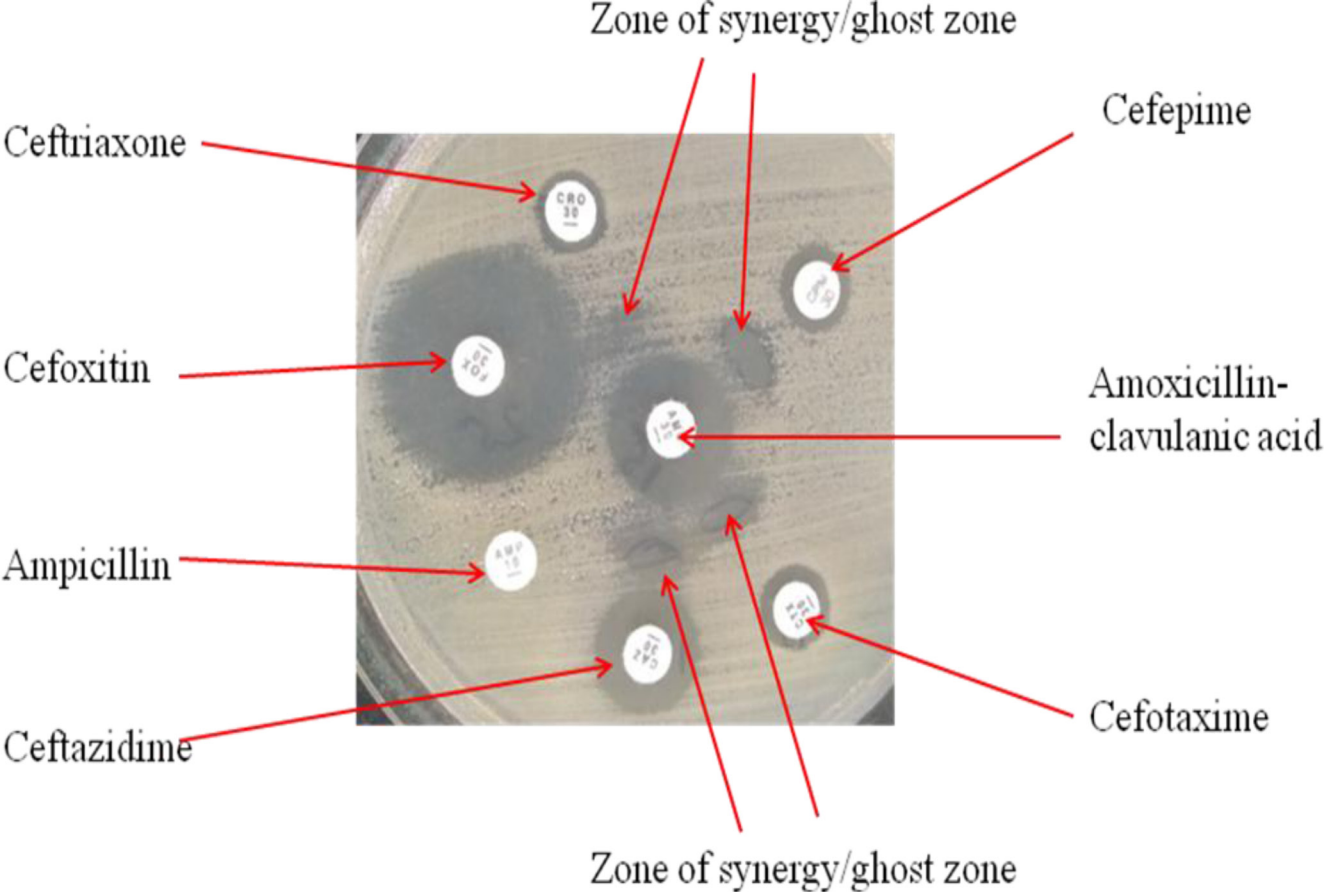
*Escherichia coli* producing extended-spectrum β-lactamases; shows an extended-spectrum β-lactamases producing *Escherichia coli* phenotype characterized by resistance towards penicillin (ampicillin) and third-generation cephalosporins (CAZ, CRO and CTX). The isolate was also resistant to more advanced fourth-generation cephalosporin (cefepime). There were synergistic reactions indicated by the ghost zones between cephalosporins and amoxicillin-clavulanic acid.

Antimicrobial susceptibility testing (AST) was done for all isolates against significant classes of antimicrobials (Oxoid, UK) on Mueller–Hinton agar (Oxoid, UK) using the disc diffusion technique (Table 1). Emulsifying pure bacterial colonies in 3 ml sterile normal saline made a 0.5 turbidity McFarland standard. A sterile cotton swab was immersed in the 0.5 McFarland suspension and spread on Mueller Hinton agar plate to ensure confluent growth. Antimicrobial discs (Oxoid, UK) were placed using a disc dispenser and plates incubated at 35 °C for 12–18 h. The zone of inhibition was measured in millimetres across the diameter. A commercial *E. coli* American-type culture collection (ATCC 25922) strain was used for quality assurance of the media and antimicrobial disc potency. The measured zones of inhibition interpretation were based on the Clinical and Laboratory Standards Institute (CLSI) 2017 guidelines [20]. Isolates resistant to ampicillin and any or all third-generation cephalosporins were presumed as extended-spectrum β-lactamases producers. Confirmation of presumed ESBL phenotype was done by double-disc synergy test. This was done by placing CAZ, CRO and CTX 30 mm apart from centrally placed AMC [21]. A positive test was indicated by the presence of a synergy zone between cephalosporin and clavulanic inhibitor (Fig. 2). Isolates with combined resistance to β-lactams, fluoroquinolone, and a broad-spectrum class of aminoglycosides such as gentamicin were denoted as βFQA-strains. In contrast, those that were resistant to ≥3 classes of antimicrobial were indicated as multidrug-resistance (MDR) strains.

**Table 1.** Antimicrobial agents tested against Gram-negative isolates in this study; this table shows the 13 antimicrobial agents belong to different classes tested against recovered Gram-negative bacteria isolates. Using the Kirby Bauer technique, the disc diffusion method was used in this antimicrobial sensitivity testing (AST)

Antimicrobial agents	Acronym	Disc potency	Manufacturer
Penicillin			
Ampicillin	AMP	10 µg	Oxoid
Third-generaion cephalosporin			
Ceftazidime	CAZ	30 µg	Oxoid
Ceftriaxone	CRO	30 µg	Oxoid
Cefotaxime	CTX	30 µg	Oxoid
Fourth-generaion cephalosporin			
Cefepime	FEP	5 µg	Oxoid
Monobactam			
Aztreonam	ATM	30 µg	Oxoid
Floroquinolone			
Ciprofloxacin	CIP	5 µg	Oxoid
Quinolone			
Nalidixic acid	na	30 µg	Oxoid
Aminoglycosides			
Gentamicin	GEN	10 µg	Oxoid
Streptomycin	S	10 µg	Oxoid
Phenicol			
Chloramphenicol	CHL	30 µg	Oxoid
Sulfomides			
Sulfamethoxozole	SMX	300 µg	Oxoid
Trimethoprim	TMP	5 µg	Oxoid
Cephamycin			
Cefoxitin	FOX	30 µg	Oxoid
Inhibitor (Agumentin)			
Amoxicillin-clavulanic acid	AMC	110 µg	Oxoid
Carbapenem			
Imipenem	IPM	10 µg	Oxoid

### Screening for β-lactam antimicrobial resistance genes (ARG)

A total of 93 isolates that were resistant to multiple-drugs (MDR) were selected for the screening of carriage of ARG and integrons. DNA extraction was done using the boiling method as previously described [22]. Briefly, a loopful of pure culture was emulsified in 1 ml distilled DNase/RNase-free water and boiling done at 95 °C for 15 min in a heating block. Centrifuging for 5 min at 14 000 rotations per minute did the separation of bacterial cell debris and DNA. Extracted DNA stained with sybr green was run on gel electrophoresis to determine yield and quality as previously described [23]. This study screened for carriage of *bla*_TEM_, *bla*_CTX-M_, *bla*_SHV_, and *bla*_OXA-1_ ARGs that are heavily implicated in resistance towards β-lactams in Kenya [24]. The isolates were also screened for carriage of class I and II integrons associated with MDR strains in Gram-negative bacteria.

The polymerase chain reaction (PCR) preparation consisted of 12 µl master mix (Sigma-Aldrich) (DNA polymerase, dNTPs, MgCL_2_ and buffer), 12 µl DNase free PCR water, and 1 µl of forward and 1 µl of reverse primers previously published (Table 2) [25–28]. Amplification was done under the following conditions; initial denaturation at 95 °C for 2 min, annealing at 50–62 °C for 1 min (depending on the target gene and the primer), extension at 72 °C for 1 min for 30 cycles, and a single final extension step at 72 °C for 15 min.

**Table 2.** PCR amplification primers used Temoneria β-lactamase, *bla*_CTX-M_: Cefotaxime Munich β-lactamase, *bla*_SHV_: Sulfhydryl β-lactamase*, Intl-*1: integrase of class one integrons, bp: expected band size in base pairs

Target gene	Primer name	Primer sequence	Annealing tºc	Product size	Reference
*bla* TEM	TEM-F	5′-GCGGAACCCCTATTTG-3′	50	964 bp	[25]
	TEM-R	5′-TCTAAAGTATATATGAGTAAACTTGGTCTGAC-3′			
*bla* CTX-M	CTX-M-F	5′-ATGTGCAGYACCAGTAARGTKATGGC-3′	60	593 bp	[25]
	CTX-M-R	5′-TGGGTRAARTARGTSACCAGAAYCAGCGG-3′			
*bla* SHV	SHV-F	5′- TTCGCCTGTGTATTATCTCCCTG- 3′	50	854 bp	[25]
	SHV-R	5′- TTAGCGTTGCCAGTGYTCG- 3′			
*bla*_OXA_	OXA-IF	5′-ATGAAAAACACAATACATATCAACTTCGC- 3′	62	820 bp	[25]
	0XA-1R	5′- GTGTGTTTAGAATGGTGATCGCATT-3′			
*Int*I1	intM1_D	5′-GAAAGGTCTGGTCATACATG-3′	50	500 bp	[28]
	intM1_U	5′-ACGAGCGCAAGGTTTCGGT-3′			
*Int*I2	INT2-L	5′-CACGGATATGCGACAAAAAGGT-3′	50	789	[27]
	INT2-R	5′-GTAGCAAACGAGTGACGAAATG-3′			
(GTG)_5_	(GTG)_5_	5′-GTGGTGGTGGTGGTG- 3′	40	variable	[26]

### Assessment of bacterial diversity

The (GTG)_5_-PCR fingerprinting method was used to evaluate bacterial diversity and possible genetic similarities among isolates of the same species using primers listed in Table 2 as described in a previous study [26]. The GTG_5_ is a method of repetitive extragenic palindromic PCR that amplifies the GTGGTGGTGGTGGTG (GTG_5_) repetitive segments present in the bacterial genome [29]. This molecular tool is useful in the differentiation of bacterial strains of the same species. The oligonucleotide primer’s binding enables the amplification of DNA fragments of varied sizes detected as multiple bands on a gel electrophoresis image.

*E. coli* and *K. pneumoniae* are good indicators of community antimicrobial resistance and were thus used to predict recovered isolates’ genetic diversity. Therefore, selected *E. coli* (8) and *K. pneumoniae* (11), resistant to at least three antimicrobial classes, were analysed to establish diversity and possible genetic relatedness. PCR amplification for the (GTG)_5_-PCR protocol was conducted under the following conditions: initial denaturation at 95 °C for 5 min, annealing at 40 °C 1 min, extension at 65 °C for 8 min for a total of 30 cycles, and a single final extension step at 65 °C for 8 min.

### Analysis of PCR amplicons

PCR amplicons were separated by running in agarose gel at 100 volts for 1 h (1% for GTG_5_-PCR and 1.2 % for ARG PCR). Visualization of banding patterns was done using a Gelmax UV imager. Cluster analysis of GTG_5_ banding patterns was done using bionumeric software version 6.6 (Gelcompar2 software). Similarity based on banding patterns was set at a threshold of 95 %, as previously described [30].

## Results

### Microbial food contamination

Analysis of microbial contamination detected levels exceeding 2.0×10^4^ c.f.u. ml^−1^ in 106 (38 %) of the 281 samples of ready-to-consume food. The mean c.f.u. for all foods was 4.9×10^5^ c.f.u. ml^−1^. More than 50 % of the food samples had a c.f.u. ml^-1^ count of between 3.7×10^5^ to 2.1×10^6^ c.f.u. ml^−1^. These values are beyond the ≤10^2^ c.f.u. ml^−1^ minimum thresholds for fit human food contamination based on HACCP standards. Among these foods, kale was the most contaminated with a mean of 2.3×10^6^ c.f.u. ml^−1^, followed by meats at 1.0×10^6^ c.f.u. ml^−1^, while Omena (a preparation of *Rastrineobola argetea*) was the least contaminated with a mean c.f.u. ml^-1^ of 4.0×10^4^ (Table 3).

**Table 3.** Food microbial load in colony-forming unit per millilitre (c.f.u. ml^−1^). Githeri is a staple food in Kenya consisting of boiled beans and corn, while Ugali is a stiff porridge prepared by mixing cornflour with boiling water before simmering. Silver cyprinids (*Rastrineobola argetea*) are a type of tiny fish commonly known as Omena in Kenya. Vigna radiate, which is popularly known by its local name Ndengu in Kenya, is a legume type of lentils while Managu (*Solanum villosum*) is a bitter type of vegetable of the nightshade family. In this table, ‘n’ is the number of food analysed, which totals to 281, CFU_50_: is the median c.f.u. ml^−1^ value, CFU_90_ is the 90th percentile, while c.f.u. ml^−1^ mode is the most common c.f.u. ml^−1^ value in the respective food vending point proximity category

Food category	Food type		Enterobacteriaceae food contamination (c.f.u. ml^−1^)		
		N	Mean	CFU_50_	CFU_90_
Vegetables					
	Kale	31	2.3×10^6^	2.3×10^6^	5.0×10^6^
	Managu	25	1.0×10^5^	1.3×10^5^	3.7×10^6^
	Cabbage	25	1.3×10^5^	1.1×10^5^	1.7×10^5^
Meat					
	Beaf	41	1.0×10^6^	1.0×10^6^	4.7×10^6^
	Omena	17	4.0×10^4^	3.0×10^4^	1.2×10^5^
Cereals					
	Beans	32	3.4×10^5^	3.0×10^5^	6.6×10^5^
	Rice	28	3.1×10^5^	2.1×10^5^	3.0×10^6^
	Githeri	36	2.0×10^5^	1.1×10^5^	3.0×10^6^
	Ndengu	24	7.0×10^4^	7.2×10^4^	1.5×10^5^
Others					
	Ugali	22	2.3×10^5^	2.4×10^5^	4.0×10^5^

### Microbial food contamination in relation to vending point proximity to surrounding features

**Fig. 3.**
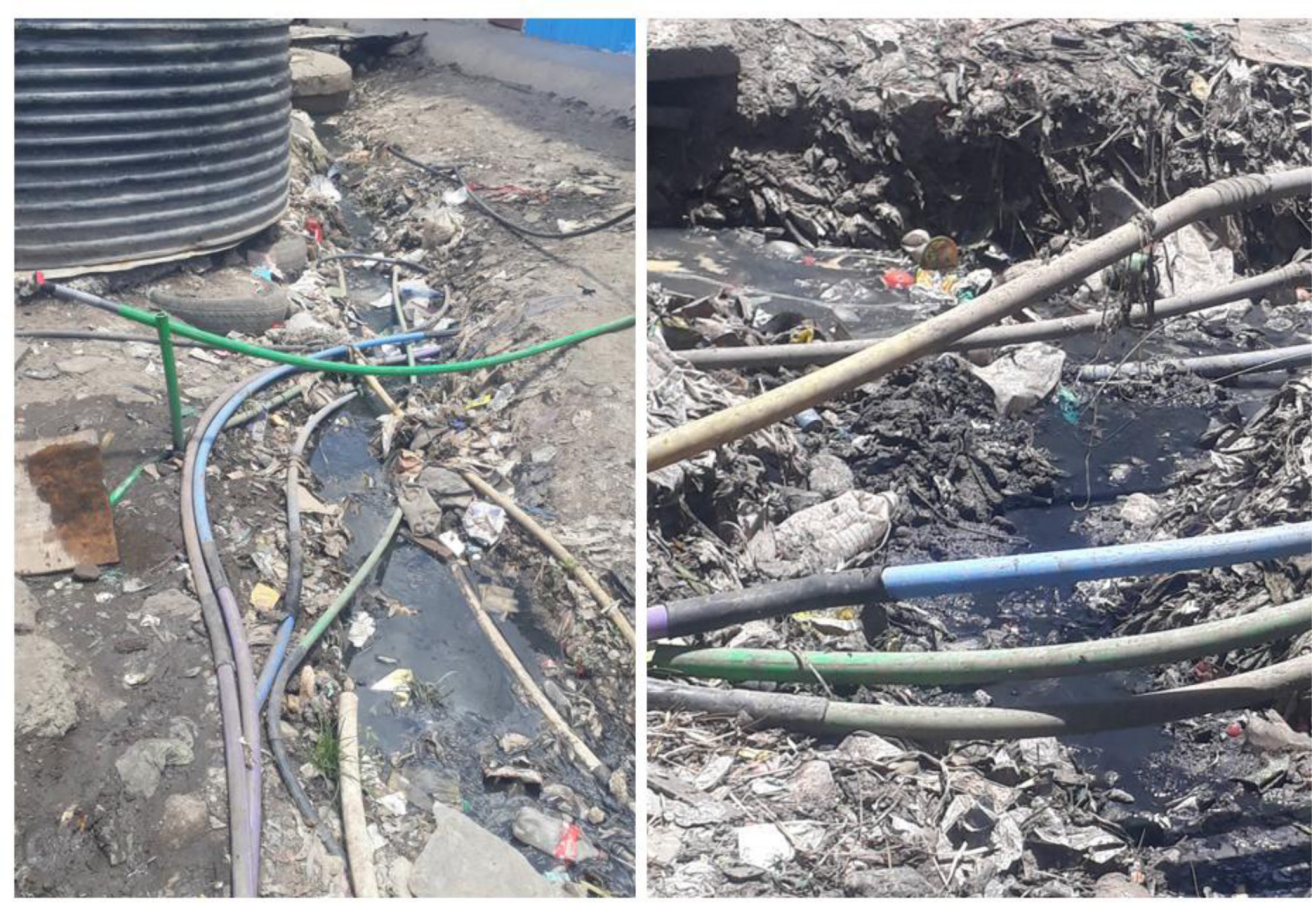
Clean water pipes laid in an open sewage and highly polluted environment in Kibera informal housing. This image was taken in Kibera informal housings and shows refuse and raw sewage at the central communal water point. Clean water supply pipes are also laid in an open sewer and refuse, which pose a risk of contamination when there are leaks and through pipe joints. Therefore, there is a high possibility that the water supply in this informal settlement is already contaminated and is a source of microbial contaminants.

This study found that foods sold close to sewage and refuse sites were the most contaminated, with a mean c.f.u. ml^−1^ of 5.5×10^5^ and 5.1×10^5^, respectively (Table 4). Statistical analysis revealed a significant difference in contamination levels among foods sold near open sewage, toilets, open space vending point layout, and near refuse site from those not sold close to these features (*P* <0.0001). Study sites that did not have a constant clean water supply were found to have more contaminated foods exceeding the recommended threshold considered fit for human consumption than those with a continuous water supply (*P* <0.0001, C.I 5.23–5.35, O.R 5.28). Fig. 3 shows an image of clean water supply lines laid on burst sewers in Kibera slums. Lack of proper personal protective equipment (PPE) and handling money significantly contributed to microbial food contamination (*P* <0.0001, C.I 0.027–0.0.028, O.R 0.028).

**Table 4.** Food contamination levels in relation to various risk factors: c.f.u. ml^−1^: colony-forming units per millilitre. Hygiene features around 10 metres radius of the food-vending point were captured, and the level of food microbial was determined in relation to proximity features analysed

Proximity of the food vending point		N	Enterobacteriaceae food contamination(c.f.u. ml^−1^)			Chi-square test			
			Mean	CFU50	CFU90	*P* value	Odds ratio (O.R)	95 % confidence interval (C.I)	
								Lower limit	Upper limit
Sewage	near	67	5.5×10^5^	7.9×10^5^	2.1×10^6^	<0.0001*	0.1183	0.1162	0.1204
	not near	33	1.4×10^4^	1.7×10^5^	2.7×10^5^				
Toilet	near	28	4.1×10^4^	9.4×10^4^	2.0×10^4^	<0.0001*	1.0278	1.0142	1.0415
	not near	72	1.3×10^5^	2.9×10^5^	3.9×10^6^				
Dump site	near	59	5.1×10^5^	1.2×10^5^	2.0×10^6^	<0.0001*	0.0235	0.0232	0.0238
	not near	41	4.0×10^4^	4.0×10^5^	6.8×10^5^				
Water supply	near	47	5.9×10^4^	1.1×10^5^	1.9×10^5^	<0.0001*	5.2825	5.2196	5.3461
	not near	53	3.4×10^5^	1.2×10^5^	2.0×10^6^				
Personal protective equipment	worn	9	2.3×10^4^	1.1×10^5^	1.8×10^5^	<0.0001*	12.8063	12.6062	13.0097
	not warn	91	2.7×10^5^	6.8×10^5^	1.6×10^6^				
Handling money while serving/preparing food	Yes	78	2.4×10^5^	3.1×10^5^	4.0×10^5^	<0.0001*	0.0283	0.0278	0.0288

*Denotes a statistically significant *P*-value, O.R: odds ratio, and C.I: confidence interval at 95 %. Association between microbial contamination means and proximity to the various environmental risk factors was calculated using Fischer’s chi-square test. In this table, the acronym ‘n’ is the number of the food vending points near or not near the analysed environmental features, totals to 100 vending points.

### Diversity of Gram-negative bacteria isolates from ready-to-eat unprocessed foods

A total of 405 microbial isolates were recovered from the 281 ready-to-eat unprocessed food samples collected from vending points (Table 5). These isolates fit into seven (7) enteric genera that namely *Klebsiella* spp (29 %), *Escherichia coli* (26 %), *Enterobacter agglomerans* (22 %), *Salmonella* spp (7 %), *Proteus mirabilis* (7 %), *Citrobacter freundii* (7 %) and *Serratia marcescens* (3 %) (Table 5). From the 405 microbial isolates, 24 % were recovered from kales, 19 % from meat, and 16 % from Managu.

**Table 5.** Diversity of bacterial isolates collected in various ready-to-eat food samples.This table shows the count of various bacterial species isolated from the ten analysed food types. The acronym spp used in this table means species

Analyszed food type	Samples analysed	No. of bacterial isolates fron the various food types						
		*Citrobacter* spp	*Enterobacter* spp	*E.coli*	*Klebsiella* spp	*Proteus* spp	*Salmonella* spp	*S. marcesence*
Kales	31	9	22	27	21	8	10	2
Meat	41	3	11	20	28	4	8	4
Beans	32	2	9	6	8	2	1	2
Rice	28	1	5	7	4	0	0	0
Ugali	22	0	3	5	8	0	0	0
Githeri	36	7	6	15	7	5	0	2
Cabbage	25	1	3	5	4	2	0	0
Managu	25	4	13	11	24	5	6	1
Ndengu	24	1	10	5	7	2	0	1
Omena	17	0	7	4	5	0	2	0
	281	28	89	105	116	28	27	12

### Antimicrobial resistance abundance in bacterial isolates

Out of the 405 Gram-negative isolates recovered from foods, the prevalence of multidrug-resistant strains was 23 % (93 isolates), 4 % (17 isolates) for ESBL strains, and 2 % (10 isolates) for βFQA-phenotype. The βFQA phenotype was prevalent in *Klebsiella* spp (4%) followed by *E. coli* (3 %) and was absent in *Citrobacter, Proteus, Salmonella, Serratia* genera. *Klebsiella* spp were the most resistant to any set of antimicrobial agents tested, with the highest values recorded towards ampicillin (AMP, 41%), trimethoprim (TMP 32 %), and sulfamethoxazole (SMX, 2 9%) (Table 6). These *Klebsiella* spp also had the highest resistance towards broad-spectrum cephalosporins (CAZ, 4 % and FEP, 3%), aminoglycoside (GEN, 6 %) and quinolones NAL (14 %) (Fluoroquinolone; CIP, 4%). However, none of the *C. freundii, Salmonella* spp*,* and *Sr. marcescens* isolates were resistant to tested cephalosporins, gentamicin or ciprofloxacin, indicating that these genera are relatively susceptible to locally available drugs and that the βFQA phenotype is still not prevalent among these genera. Resistance patterns close to those recorded in *K. Pneumoniae,* was noted in *E. coli* isolates (CAZ 2 %, FEP 2 %, GEN 5 %, NAL 10 %, and CIP 3%) (Table 6). All of the 405 isolates recovered in this study were susceptible to imipenem (IPM, 100 %) while 98 % were susceptible to cefepime, a fourth-generation cephalosporin.

**Table 6.** Relative abundance (%) of isolates antimicrobial resistant from ready-to-eat foods: non-duplicate colonies from the primary plate were subjected to susceptibility testing against some of the most commonly used antimicrobial agents. Antimicrobial activity of these isolates was tested against 15 drugs belonging to penicillin, β-lactam, fluoroquinolone, quinolone, aminoglycoside, carbapenem, and phenicol and sulfonamide classes. The disc diffusion method was used in this sensitivity testing using inoculums of 0.5 McFarland standards

Gram-negative bacteria	N	Antimicrobial resistance profiles of Gram-negative microbials (%)														
		AMP	CTX	CAZ	CRO	FEP	ATM	FOX	AMC	GEN	S	CIP	NAL	IPM	SMX	TMP
All	405	133 (33)	17 (4)	7 (2)	17 (4)	7 (2)	21 (5)	46 (11)	39 (10)	15 (4)	40 (10)	10 (2)	36 (9)	0(0)	95 (23)	110 (27)
* C. freundii *	27	7 (26)	0 (0)	0 (0)	0 (0)	0 (0)	0 (0)	1 (4)	1 (4)	0 (0)	1 (4)	0 (0)	1 (4)	0 (0)	5 (19)	6 (22)
* E. agglomerans *	88	24 (27)	3 (3)	0 (0)	3 (3)	1 (1)	4 (5)	9 (10)	7 (8)	2 (2)	7 (8)	2 (2)	7 (8)	0 (0)	20 (23)	22 (25)
* E. coli *	104	34 (33)	5 (5)	2 (2)	5 (5)	2 (2)	6 (6)	14 (13)	11 (11)	5 (5)	12 (12)	3(3)	10 (10)	0 (0)	25 (24)	29 (28)
*Klebsiella* spp	116	47 (41)	8 (7)	5 (4)	7 (6)	4 (3)	9 (8)	17 (15)	15 (13)	7 (6)	15 (13)	5 (4)	14 (12)	0 (0)	34 (29)	37 (32)
* P. mirabilis *	30	9 (30)	1 (3)	0 (0)	1 (3)	0 (0)	1 (3)	2 (7)	2 (7)	1 (3)	2 (7)	0 (0)	2 (7)	0 (0)	5 (17)	7 (23)
*Salmonella spp*	28	9 (32)	0 (0)	0 (0)	1 (4)	0 (0)	1 (4)	2 (7)	2 (7)	0 (0)	2 (7)	0 (0)	1 (4)	0 (0)	4 (14)	7 (25)
*Sr. marcesence*	12	3 (25)	0 (0)	0 (0)	0 (0)	0 (0)	0 (0)	1 (8)	1 (8)	0 (0)	1 (8)	0 (0)	1 (8)	0 (0)	2 (17)	2 (17)

Among the ten food types collected and analysed in this study, kale followed by meat isolates were the most resistant overall. In contrast, rice, Ndengu, and Ugali isolates were the least resistant (Table 7). The analysis also showed that isolates from kale samples were also the most resistant to broad-spectrum antimicrobial agents; AMC 13 %, GEN 7 %, CIP 6 %, FEP 4 %, and CAZ 4 % (Table 7).

**Table 7.** Relative abundance in antimicrobial resistance among isolates recovered from the various foods types: the table shows the antimicrobial resistance abundance of various isolates recovered from the ten food sample types collected in this study. The acronym ‘n’ used in this table indicates the count of bacterial isolates recovered from each of the ten food types collected and analysed in this study

Food type	N	AMP	CTX	CAZ	CRO	FEP	ATM	FOX	AMC	GEN	S	CIP	NAL	IPM	SMX	TMP
Kales	99	55 (56)	7 (7)	4 (4)	7 (7)	4 (4)	9 (9)	17 (17)	13(13)	7 (7)	13 (13)	6 (6)	11 (11)	0 (0)	32 (32)	45 (45)
Meat	78	32 (41)	4 (5)	2 (3)	4 (5)	2 (3)	4 (5)	9 (12)	7 (9)	1 (1)	9 (12)	3 (4)	6 (8)	0 (0)	14 (18)	23 (29)
Beans	30	10 (33)	1 (3)	0 (0)	1 (3)	0 (0)	1 (3)	3 (10)	3 (10)	1 (3)	3 (10)	0 (0)	5 (17)	0 (0)	11 (37)	5 (17)
Rice	17	4 (24)	0 (0)	0 (0)	0 (0)	0 (0)	1 (6)	2 (12)	2 (12)	0 (0)	1 (6)	0 (0)	2 (12)	0 (0)	5 (29)	3 (18)
Ugali	16	5 (31)	0 (0)	0 (0)	0 (0)	0 (0)	0 (0)	2 (13)	1 (6)	0 (0)	1 (6)	0 (0)	1 (6)	0 (0)	4 (25)	5 (31)
Githeri	42	10 (24)	2 (5)	0 (0)	2 (5)	0 (0)	2 (5)	5 (12)	6 (14)	2 (5)	5 (12)	0 (0)	4 (10)	0 (0)	7 (17)	7 (17)
Cabbage	15	4 (27)	0 (0)	0 (0)	0 (0)	0 (0)	0 (0)	2 (13)	1 (7)	0 (0)	1 (7)	0 (0)	1 (7)	0 (0)	3 (20)	4 (27)
Managu	64	4 (27)	3 (5)	1 (2)	3 (5)	1 (2)	3 (5)	4 (6)	4 (6)	2 (3)	4 (6)	1 (2)	4 (6)	0 (0)	9 (14)	9 (14)
Ndengu	26	4 (15)	0 (0)	0 (0)	0 (0)	0 (0)	1 (4)	1 (4)	1 (4)	0 (0)	2 (8)	0 (0)	1 (4)	0 (0)	5 (19)	5 (19)
Omena	18	5 (28)	0 (0)	0 (0)	0 (0)	0 (0)	0 (0)	1 (6)	1 (6)	0 (0)	1 (6)	0 (0)	1 (6)	0 (0)	5 (28)	4 (22)
All	405	133 (33)	17 (4)	7 (2)	17 (4)	7 (2)	21 (5)	46 (11)	39 (10)	13 (3)	40 (10)	10 (2)	36 (9)	0 (0)	95 (23)	110 (27)

### PCR analysis of β-lactam ARG and integrons

**Fig. 4.**
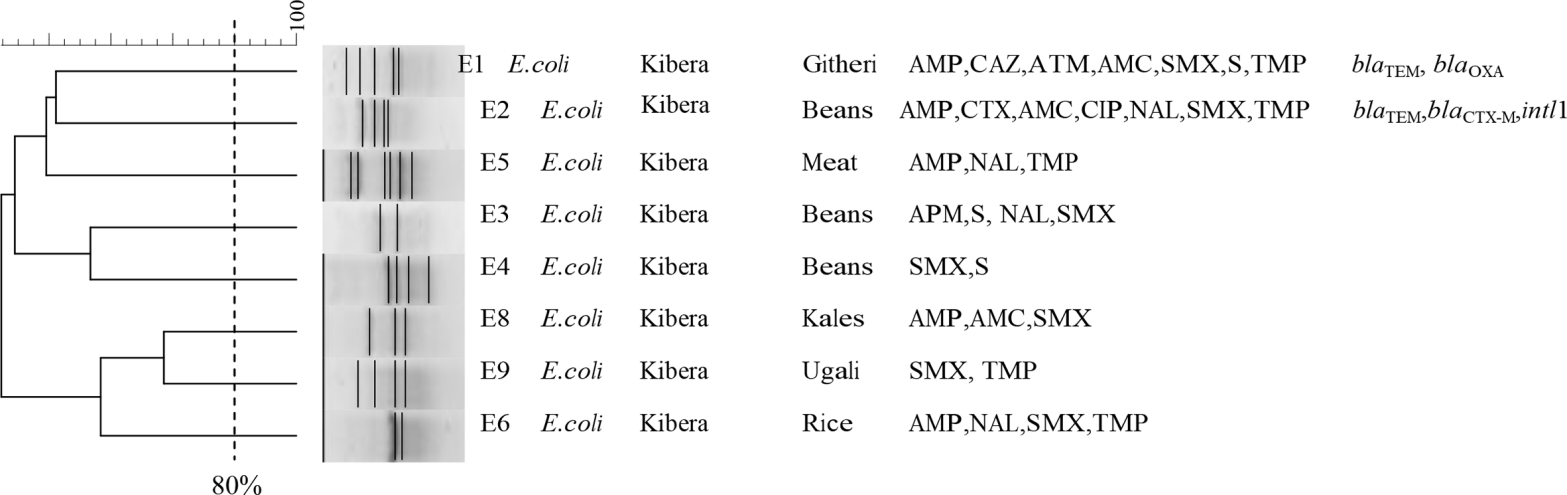
Cluster analysis of *Escherichia coli* isolates from ready-to-eat foods. This dendrogram shows the clustering patterns of *Escherichia coli* isolates from ready-to-eat unprocessed food samples. The first column on this image represents the unique identification number of the analysed isolates, while the third, fourth and fifth are the food types, antimicrobial agents, and resistance genes, respectively. Drawing a vertical straight line at an 80 % mark on the calibrations above the dendrogram assessed similarity index among isolates that cluster together. The acronym ‘E’ followed by a digit represents *Escherichia coli* unique identification. Other acronyms used in this figure includes; AMP: Ampicillin, ATM: Aztreonam, AMC: Amoxicillin-clavulanic acid, CAZ: Ceftazidime, CTX: cefotaxime, NA: Nalidixic acid, SMX: sulfamethoxazole, TMP: Trimethoprim, TEM: Temoneria β-lactamase gene, SHV: Sulfhydryl variable β-lactamase gene, CTX-M: Cefotaxime Munich β-lactamase gene, *intl*1: integrin of class one integrons.

Among the 93 MDR-strains screened for carriage of ESBL genes, *bla*_TEM_ was the most prevalent (51, 55 %), followed by *bla_OXA_*_-1_ (36, 39 %), *bla*_CTX-M_ (nine, 10 %), and *bla*_SHV_ (seven, 8 %) (Table 8). Among the 17 presumed ESBL-producing strains (based on phenotypic resistance to all/either CRO, CTX, and CAZ), *bla*_TEM_ was the most common (17, 100 %) followed by *bla*_OXA-1_, (12, 71 %). Prevalence of *bla* genes prevalent in *K. pneumoniae* and *E. coli* was *bla*_TEM_ (76, 63%) *bla*_CTX-M_ (12, 15 %) and *bla*_OXA_ (45, 41 %) respectively. Of the 39 isolates resistant to AMC, 36 (92 %) carried the *bla*_OXA-1_ gene that encodes amoxicillin-clavulanic acid (AMC) resistance. Class one integrons were more prevalent (21, 23 %) than *intI*2 (three, 3 %) among these MDR-strains. Prevalence of *intI*1 and *intI*2 was more prevalent in the βFQA- strains compared to ESBL-producers, *intI*1 (five, 50 %; eight, 47 %) and *intI*2 (two, 20 %; three 17 %) respectively. The distribution pattern of isolates positive for the carriage of integrons is shown in Fig. 4.

**Table 8.** Carriage of β-lactamase genes and integron class 1 in Gram-negative microbials: PCR method was used to detect carriage of β-lactamases and classes 1 and 2 integrons (*intl*1, *intl*2) in 93 Gram-negative bacteria resistant to three or more antimicrobial agents that belong to different classes

Microbial isolates	Screened isolates	Carriage of β-lactamases genes and integron in Gram-negative microbials					
		TEM	CTX-M	SHV	OXA-1	int1	Int2
* C. freundii *	5	1 (20)	0 (0)	0 (0)	1 (20)	0 (0)	0 (0)
* E. agglomerans *	14	5 (36)	1 (7)	0 (0)	7 (50)	3 (21)	0 (0)
* E. coli *	27	17(63)	4 (15)	2 (7)	11 (41)	7 (26)	1 (4)
*Klebsiella* spp	33	25(76)	4 (12)	5 (15)	15 (45)	10 (30)	2 (6)
* P. mirabilis *	5	2 (40)	0 (0)	0 (0)	1 (20)	1 (20)	0 (0)
*S. Tyhimurium*	6	1 (17)	0 (0)	0 (0)	1 (17)	0 (0)	0 (0)
*Sr. marcesence*	3	0 (0)	0 (0)	0 (0)	0 (0)	0 (0)	0 (0)
MDR-strains	93	51 (55)	9 (10)	7 (8)	36 (39)	21 (23)	3 (3)
ESBLs strains	17	17 (100)	9 (53)	7 (41)	12 (71)	8 (47)	3 (17)
βFQA strains	10	8 (80)	9 (90)	7 (70)	7 (70)	5 (50)	2 (20)

### Analysis of clustering patterns in selected bacteria species

**Fig. 5.**
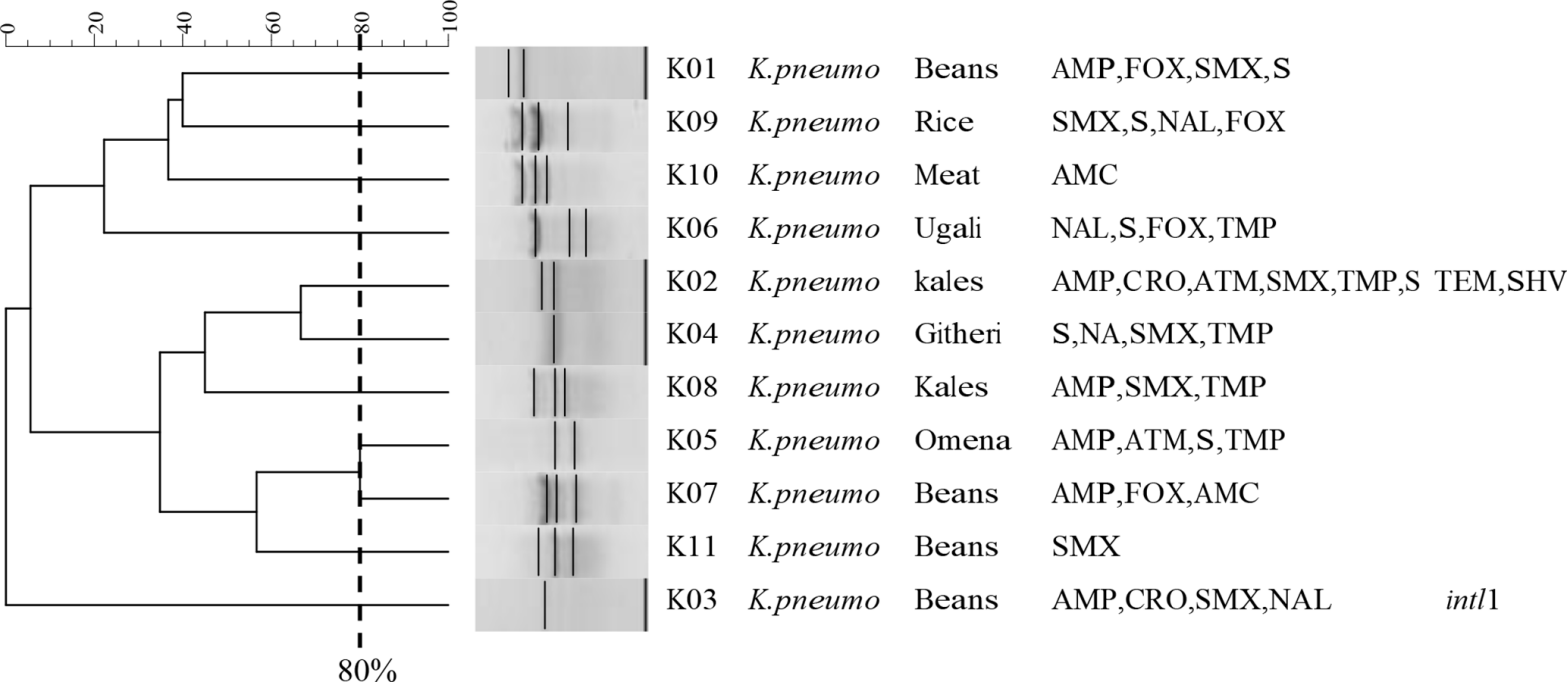
Cluster analysis of *K. pneumoniae* isolates from ready-to-eat foods. Twelve *Klebsiella pneumoniae* isolates were selected based on food type recovered from and resistance phenotypes. The dendrogram was generated based on banding using the Gelcompar Bionumeric software. The acronym ‘K’ followed by a digit represents *Klebsiella pneumoniae* unique identification. Other acronyms used in this figure includes; AMP: Ampicillin, ATM: Aztreonam, AMC: Amoxicillin-clavulanic acid, CAZ: Ceftazidime, CTX: cefotaxime, NA: Nalidixic acid, SMX: sulfamethoxazole, TMP: Trimethoprim, TEM: Temoneria β-lactamase gene, SHV: Sulfhydryl variable β-lactamase gene, *intl*1: integrin of class one integrons.

A similarity matrix of less than 40 % was noted among *ndicating low genetic similarity. Tw. coli* isolates obtained from various food types across Kibera slums (Fig. 4). The analysis revealed no evidence of the proliferation of specific *E. coli* clones. *E.coli* isolates in this dendrogram were resistant to ampicillin, trimethoprim, and sulfamethoxazole in addition to other varying antimicrobial agents, indicating low genetic similarity. Two *K. pneumoniae* from Omena (Silver cyprinid) and beans foods had a similarity of 80 % likely suggesting microbial cross-contamination (Fig. 5). Most isolates in the *K. pneumoniae* dendrogram had a similarity range of 40–80 %, and 50 % of these isolates were resistant to ampicillin, trimethoprim, and sulfamethoxazole.

## Discussion

The mean contamination of between 4.0×10^4^ c.f.u. ml^−1^ and 2.3×10^6^ c.f.u. ml^−1^ among food analysed in this study are close to the 6.7×10^5^ to 1.7×10^6^ c.f.u. ml^−1^ recorded for fish foods in Ethiopia [31]. Among the cereal-type foods, the highest contamination was recorded in beans with a mean of 3.4×10^5^ c.f.u. ml^−1^, which is lower than 8.1×10^3^ c.f.u. ml^−1^ reported in maize/beans (Githeri) in Mukuru slum in Kenya [32]. Vegetables such as kale were the most contaminated food type with a mean value of 2.3×10^6^ c.f.u. ml^−1^, close to 6.8×10^6^ c.f.u. g^−1^ recorded in South Africa among the same food type [33]. These findings affirm the World Health Organization (WHO) 2008 Report which indicated that leafy vegetables pose a higher risk of foodborne infection in humans. Microbial contamination of vegetables has previously been attributed to unsafe urban irrigation using sewage water often contaminated with human excreta [34]. This study ratifies that most street foods sold in Kibera are highly contaminated, which is likely to increase diarrhoea cases and other gastrointestinal infections [32, 35]. We also recovered some pathogenic strains such as *Salmonella* spp that have a low infection dose, which further increases the risk of foodborne infections. Notably, diarrhoea is a significant cause of morbidity and mortality in developing countries, with *Shigella, E. coli,* and *Salmonella* genera documented as the primary etiological agents in slums such as Kibera [36].

A significant association in microbial contamination to the immediate unhygienic vending environment was noted. A similar study in Kenya has also indicated that foods sold close to refuse sites and burst sewers are more contaminated, which calls for improved sanitation and hygiene standards [37]. We also noted that most vendors lack a constant supply of clean water for washing raw foods and utensils. Poor sanitation and chronic water shortages have been identified as significant contributors to enteric infections in slums such as Kibera [38]. Similarly, our previous study reported a diverse range of MDR bacteria belonging to *Salmonella* spp*, Shigella* spp*, E. coli,* and *Klebsiella* spp from sewage, sludge, and soil in Kibera near these food vending points [39]. Therefore, there is an excellent possibility that most of the enteric isolates among these foods emanate from unhygienic surroundings. Although we did not screen for virulence genes in recovered isolates, consumption of foods contaminated with *Salmonella* spp may particularly have the potential to cause gastrointestinal infections and death. Notably, *Salmonella* spp in this study were recovered without the usual 24 h enrichment in modified Rappaport Vassiliadis protocols recommended for *Salmonella* spp recovery from contaminated food and therefore, it is possible that occurrence of this pathogen in our food specimen could be higher than that reported.

Our isolates were more resistant to ampicillin, trimethoprim, and sulfamethoxazole. This finding is similar to previous findings in Kenya that recorded 34 % resistance towards AMP and 49.4 % towards sulfamethoxazole-trimethoprim among *E. coli* isolates from raw chicken [40]. Therefore, it is likely that bacteria strains circulating in Kenya have built resistances towards these antimicrobial agents; however, these assumptions need to be ascertained through further research. Compared to Odwar *et al.* (2014), our study recorded higher resistances towards amoxicillin-clavulanic acid (AMC 11 % vs. 2.6 %), ceftazidime (CAZ 2 % vs. 0 %), and gentamicin (GEN 5 % vs. 0.6 %). A similar study in Ethiopia has documented higher resistances of up to 80 % for ampicillin, 14.3 % for ceftriaxone and 9.5 % for gentamicin in *E. coli* from fish [31]. The variation in antimicrobial resistance abundance strongly indicates microbial isolates in these regions are evolving and developing antimicrobial resistance independently and is likely to reflect a pattern of antimicrobial usage and AMR burden, but these assumptions remain to be elucidated as well. Our recent study among Gram-negative isolates from sewage, sludge, and soil recovered near these vending points revealed high resistances towards ceftazidime (9 %), ceftriaxone (12 %), amoxicillin-clavulanic acid (16%), gentamicin (11%) and ciprofloxacin (8%) [39]. Taken together, these results could be an indication that the resistant strains may be emanating from the immediate surroundings to contaminate slum foods. Resistances of up to 2 % towards ceftazidime, ciprofloxacin, and 9 % against aztreonam have been documented in *Klebsiella* species from stool samples in Kenya [41]. These resistances are similar to those recorded in food isolates in our study and also suggest a high possibility that such isolates may also have a clinical origin. Findings in this study indicated that carbapenem (imipenem) is still effective against recovered isolates (100 %). However, co-resistance to β-lactams, aminoglycosides, and fluoroquinolone, commonly available and used antimicrobial agents in Kenya, is worrying [42]. There is a great risk of treatment failure of infections caused by the ESBL and βFQA phenotypes emanating from the consumption of foods contaminated with such enterics [43].

In the current study, the prevalence of class integrons was higher than that of *intI*2 in Gram-negative isolates, and this correlated with the findings of another study in Iran [44]. Although we did not establish recovered integrons’ resistance cassette content, the *drfA*1-*sat*2-*aad*A1, *sat*2, *sat*1-aadA1 cassettes in class two integrons have previously been associated with resistance towards sulfonamides and β-lactams [45]. Most isolates that carried class two (*intI*2) integrase were also positive for *intI*1 carriage. Therefore, bacterial strains that carry these integrons have a high potential to capture, accumulate, and express ARG leading to AMR emergence. If these integrons are contained in mobile plasmids, these resistance genes can be spread between and across species, consequently worsening AMR’s menace. A study in South Africa has also reported carriage of *bla*_CTX-M_, *bla*_TEM_, *bla*_SHV_, *bla*_OXA_ and *intI*1 in *E. coli* and *K. pneumoniae* isolates from spinach samples [46]. These β-lactams genes code for antimicrobial resistance enzymes that have been associated with resistances in diverse β-lactam antibiotics such as ampicillin, ceftriaxone, cefotaxime and ceftazidime [14].

Fingerprint analysis revealed no significant clones of *E. coli* and *K. pneumonia*. These findings suggest that the MDR, βFQA, and/or ESBL phenotypes may be spreading through horizontal or vertical gene transfer mechanisms rather than through the expansion of significant clones. However, a few isolates from different food types had similar resistances suggesting that major food types may have been contaminated from a common source, which we suspect to be the water used for cooking and food preparation.

## Conclusion

This study’s high relative microbial contamination indicates that many foods served and prepared in Kibera Street are unsafe for human consumption. Therefore, affirmative action needs to be taken by public health officials to ensure food safety awareness and hygiene. Deliberate efforts geared towards improved sanitation infrastructure such as organized refuse collection and proper sewage drainage also need to be enforced. Future studies in the informal settlements should also establish the possible origins of multidrug-resistance strains, especially in hotspot areas identified by this study.

### Study limitations

1. This study could only screen for the carriage of selected β-lactamase genes; therefore, the full array of antimicrobial resistance genes in recovered isolates is unknown. The whole array of antimicrobial resistance genes can help justify the observed resistance phenotypes.
2. The GTG_5_ low resolution fingerprinting technique was used to establish the diversity of recovered bacterial isolates. Therefore, future studies should use high-resolution techniques such as SNP typing and whole-genome sequencing to provide insight into recoverable isolates' genetics. Such methods could shed more light on the isolates' transmission pathways, the potential for isolates as pathogens, and the profound molecular basis of resistance.
3. While sample collection was done in 2017, findings dissemination was done in 2021; the data should be updated by conducting frequent surveys. However, data generated in this study forms a basis for robust future studies.

## References

[1] Alegbeleye OO, Singleton I, Santgcona AS (2018). Sources and contamination routes of microbial pathogens to fresh produce during field cultivation: A review. Food Microbiol.

[2] Machado-Moreira B, Richards K, Brennan F, Abram F, Burgess CM (2019). Microbial contamination of fresh produce: what, where, and how. Compr Rev Food Sci Food Saf.

[3] Jaffee S, Henson S, Unnevehr L, Grace D, Cassou E (2018). The Safe Food Imperative: Accelerating Progress in Low-And Middle-Income Countries.

[4] Kirk MD, Pires SM, Black RE, Caipo M, Crump JA (2015). World Health Organization estimates of the global and regional disease burden of 22 foodborne bacterial, protozoal, and viral diseases, 2010: a data synthesis. PLoS Med.

[5] Alimi BA (2016). Risk factors in street food practices in developing countries: A review. Food Sci Hum Well.

[6] Sharma I, Mazumdar JA (2014). Assessment of bacteriological quality of ready to eat food vended in streets of Silchar city, Assam, India. Indian J Med Microbiol.

[7] Doyle ME (2015). Multidrug-resistant pathogens in the food supply. Foodborne Pathog Dis.

[8] Yang X, Wu Q, Zhang J, Huang J, Chen L (2019). Prevalence, bacterial load, and antimicrobial resistance of *Salmonella serovars* isolated from retail meat and meat products in China. Front Microbiol.

[9] Cheng G, Ning J, Ahmed S, Huang J, Ullah R (2019). Selection and dissemination of antimicrobial resistance in Agri-food production. Antimicrob Resist Infect Control.

[10] Lekshmi M, Ammini P, Kumar S, Varela MF (2017). The food production environment and the development of antimicrobial resistance in human pathogens of animal origin. Microorganisms.

[11] Sabbagh P, Rajabnia M, Maali A, Ferdosi-Shahandashti E (2020). Integron and its role in antimicrobial resistance; A literature review on some bacterial pathogens. Iran J Basic Med Sci.

[12] Gillings M, Boucher Y, Labbate M, Holmes A, Krishnan S (2008). The evolution of class 1 integrons and the rise of antibiotic resistance. J Bacteriol.

[13] Partridge SR, Kwong SM, Firth N, Jensen SO (2018). Mobile genetic elements associated with antimicrobial resistance. Clin Microbiol Rev.

[14] Kiiru J, Kariuki S, Goddeeris BM, Butaye P (2012). Analysis of β-lactamase phenotypes and carriage of selected β-lactamase genes among *Escherichia coli* strains obtained from Kenyan patients during an 18-year period. BMC Microbiol.

[15] Langata LM, Maingi JM, Musonye HA, Kiiru J, Nyamache AK (2019). Antimicrobial resistance genes in *Salmonella* and *Escherichia coli* isolates from chicken droppings in Nairobi, Kenya. BMC Res Notes.

[16] Pitout JDD, Revathi G, Chow BL, Kabera B, Kariuki S (2008). Metallo-β-lactamase-producing *Pseudomonas aeruginosa* isolated from a large tertiary centre in Kenya. Clin Microbiol Infect.

[17] Maina JN (2020). Mapping the distribution patterns of multiple-drug resistances gram-negative bacterial strains recoverable from food and environmental samples in Kibera informal settlements. Doctoral dissertation.

[18] Hemraj V, Diksha S, Avneet G (2013). A review on commonly used biochemical tests for bacteria. Innovare J Life Sci.

[19] Herrera AG (2004). The hazard analysis and critical control point system in food safety. In Public Health Microbiology.

[20] Wayne PA (2017). Clinical and Laboratory Standards Institute.

[21] Shaikh NK, Mundhada SG, Lalngaihzuali R, Ingole K (2016). Comparison of different phenotypic methods for the detection of extended spectrum b-lactamase (ESBL) in bacterial isolates from tertiary care centre. Int J Curr Res.

[22] Abdelhai MH, Hassanin HA, Sun X (2016). Comparative study of rapid dna extraction methods of pathogenic bacteria. Am J Biosci Bioeng.

[23] Kheyrodin H, Ghazvinian K (2012). DNA purification and isolation of genomic DNA from bacterial species by plasmid purification system. Afr J Agric Res.

[24] Mwangi NS (2016). Antimicrobial resistance patterns and genetic basis of extended spectrum β-Lactamases in faecal *Escherichia coli* isolated from severely malnourished and Non-Malnourished children attending mbagathi district hospital, Nairobi. Doctoral dissertation, jkuat.

[25] Hasman H, Mevius D, Veldman K, Olesen I, Aarestrup FM (2005). beta-Lactamases among extended-spectrum beta-lactamase (ESBL)-resistant *Salmonella* from poultry, poultry products and human patients in The Netherlands. J Antimicrob Chemother.

[26] Mohapatra BR, Broersma K, Mazumder A (2007). Comparison of five rep-PCR genomic fingerprinting methods for differentiation of fecal *Escherichia coli* from humans, poultry and wild birds. FEMS Microbiol Lett.

[27] Taherikalani M, Maleki A, Sadeghifard N, Mohammadzadeh D, Soroush S (2011). Dissemination of class 1, 2 and 3 integrons among different multidrug resistant isolates of *Acinetobacter baumannii* in Tehran hospitals, Iran. Pol J Microbiol.

[28] Yu T, Jiang X, Zhou Q, Wu J, Wu Z (2014). Antimicrobial resistance, class 1 integrons, and horizontal transfer in *Salmonella* isolated from retail food in Henan, China. J Infect Dev Ctries.

[29] Kathleen MM, Samuel L, Felecia C, Ng KH, Lesley MB (2014). GTG 5)analy5-PCR16s analands16Squrrnabacteriseqofrom sarbactefromaculSarawaknmentaquaculture. Int Food Res J.

[30] Vaez H, Moghim S, Nasr Esfahani B, Ghasemian Safaei H (2015). Clonal relatedness among imipenem-resistant *Pseudomonas aeruginosa* isolated from icu-hospitalized patients. Crit Care Res Pract.

[31] Eromo T, Tassew H, Daka D, Kibru G (2016). Bacteriological quality of street foods and antimicrobial resistance of isolates in Hawassa, Ethiopia. Ethiop J Health Sci.

[32] Muoki MA, Tumuti DS, Rombo D (2008). Nutrition and public hygiene among children under five years of age in Mukuru slums of Makadara Division. East Afr Med J.

[33] Nyenje ME, Odjadjare CE, Tanih NF, Green E, Ndip RN (2012). Foodborne pathogens recovered from ready-to-eat foods from roadside cafeterias and retail outlets in Alice, Eastern Cape Province, South Africa: public health implications. Int J Environ Res Public Health.

[34] Qadir M, Wichelns D, Raschid-Sally L, McCornick PG, Drechsel P (2010). The challenges of wastewater irrigation in developing countries. Agric Water Manag.

[35] Nyokabi S, Birner R, Bett B, Isuyi L, Grace D (2018). Informal value chain actors’ knowledge and perceptions about zoonotic diseases and biosecurity in Kenya and the importance for food safety and public health. Trop Anim Health Prod.

[36] Samuel S, Moses N, Too E (2019). Prevalence of Enterobacteriaceae Isolated from Childhood Diarrhoea in Mukuru Slums.

[37] Kariuki EN (2018). Bacteriological safety of street foods and factors associated with food contamination among street food vendors in Githurai and Gikomba markets. Doctoral dissertation, JKUAT-COHES.

[38] Olack B, Feikin DR, Cosmas LO, Odero KO, Okoth GO (2014). Mortality trends observed in population-based surveillance of an urban slum settlement, Kibera, Kenya, 2007–2010. PLoS One.

[39] Maina J, Ndung’u P, Muigai A, Onyango H, Mukaya JK (2019). Antimicrobial profiles of selected gram-negative bacteria recoverable from sewage and sludge from Juja and Kibera informal settlements of the larger Nairobi metropolis. Adv Microbiol.

[40] Odwar JA, Kikuvi G, Kariuki JN, Kariuki S (2014). A cross-sectional study on the microbiological quality and safety of raw chicken meats sold in Nairobi, Kenya. BMC Res Notes.

[41] Taitt CR, Leski TA, Erwin DP, Odundo EA, Kipkemoi NC (2017). Antimicrobial resistance of *Klebsiella pneumoniae* stool isolates circulating in Kenya. Plos one.

[42] Kiiru J, Kariuki S, Goddeeris BM, Revathi G, Maina TW (2011). *Escherichia coli* strains from Kenyan patients carrying conjugatively transferable broad-spectrum β-lactamase, qnr, aac(6’)-Ib-cr and 16S rRNA methyltransferase genes. J Antimicrob Chemother.

[43] Threlfall EJ, Ward LR, Frost JA, Willshaw GA (2000). The emergence and spread of antibiotic resistance in foodborne bacteria. Int J Food Microbiol.

[44] Tajbakhsh E, Khamesipour F, Ranjbar R, Ugwu IC (2015). Prevalence of class 1 and 2 integrons in multi-drug resistant *Escherichia coli* isolated from aquaculture water in Chaharmahal Va Bakhtiari province, Iran. Ann Clin Microbiol Antimicrob.

[45] Wu K, Wang F, Sun J, Wang Q, Chen Q (2012). Class 1 integron gene cassettes in multidrug-resistant Gram-negative bacteria in southern China. Int J Antimicrob Agents.

[46] Richter L, Du Plessis EM, Duvenage S, Korsten L (2020). Occurrence, phenotypic and molecular characterization of extended-spectrum-and AmpC-β-Lactamase producing Enterobacteriaceae isolated from selected commercial spinach supply chains in South Africa. Front Microbiol.

